# Controlled-Atmosphere Corrosion Engineering Toward NiFe-LDH Enabling High-Performance Alkaline Seawater Electrolysis with Long-Term Stability

**DOI:** 10.3390/mi17060675

**Published:** 2026-05-29

**Authors:** Yang Su, Yuqing Li, Qing Wang, Yue Hu, Liu Han, Xiyuan Feng, Bin Wu, Jie Wang, Yingtang Zhou

**Affiliations:** 1 Zhejiang Key Laboratory of Pollution Control for Port–Petrochemical Industry, Marine Science and Technology College, Zhejiang Ocean University, Zhoushan 316022, Chinawq1398903367@163.com (Q.W.);; 2 National Engineering Research Center for Marine Aquaculture, Zhejiang Ocean University, Zhoushan 316022, China; 3 School of Chemistry and Chemical Engineering, Anhui Provincial Key Laboratory of Advanced Catalysis and Energy Materials, Anhui Key Laboratory of Optoelectronic Magnetic Functional Complex and Nano Complex, Anqing Normal University, Anqing 246011, Chinawangjie@aqnu.edu.cn (J.W.); 4 School of Integrated Circuits (School of Microelectronics), Northwestern Polytechnical University, Xi’an 710129, China; 5 School of Materials Science and Engineering, Nanyang Technological University, Singapore 639798, Singapore; 6 Hubei Key Laboratory of Plasma Chemistry and Advanced Materials, Wuhan Institute of Technology, Wuhan 430205, China

**Keywords:** seawater electrolysis, NiFe-LDH, sulfur modification, room-temperature synthesis

## Abstract

Electrochemical water splitting stands as a feasible approach for sustainable hydrogen production, but its industrial implementation is restricted by sluggish oxygen evolution reaction (OER) kinetics and excessive dependence on freshwater resources. As a widely existing alternative, seawater contains a high concentration of chloride ions (Cl^−^), which give rise to serious electrode corrosion and catalyst deactivation, bringing great challenges to actual electrolysis applications. Herein, we report a facile room-temperature two-step soaking strategy to fabricate sulfur-modified NiFe layered double hydroxide (S-NiFe-LDH) catalysts for efficient OER in both alkaline freshwater and seawater electrolytes. The introduction of sulfur not only optimizes the electronic structure of NiFe-LDH to strengthen intrinsic catalytic activity and speed up charge transfer, but also promotes the formation of a Cl^−^-resistant layer, thus significantly improving corrosion resistance. In addition, DFT calculations show sulfur modification in NiFe layered double hydroxide upshifts the O 2p-band center to activate lattice oxygen, switches the oxygen evolution reaction pathway to the lattice oxygen mechanism with reduced thermodynamic barriers, and realizes the selective adsorption of OH^−^ over Cl^−^. As a result, the as-prepared S-NiFe-LDH catalyst exhibits exceptional OER performance, requiring overpotentials (η) of 250, 270, and 290 mV to reach current densities of 50, 100, and 200 mA·cm^−2^ in 1 M KOH, respectively, with a Tafel slope of 22.3 mV·dec^−1^. Moreover, it maintains remarkable stability for more than 200 h in alkaline seawater electrolytes and achieves nearly 100% Faradaic efficiency for water splitting, effectively avoiding the parasitic chlorine evolution reaction (CER). This work provides a scalable and energy-efficient synthetic route for designing advanced non-noble metal catalysts, paving the way for industrial-scale hydrogen production from seawater.

## 1. Introduction

Hydrogen energy has become a key clean energy carrier to ease global energy shortage and environmental pollution, and electrochemical water splitting is regarded as one of the most promising ways to realize sustainable hydrogen production [1,2,3,4,5,6]. However, the large-scale application of this technology is limited by two key problems: the slow reaction kinetics of the anodic oxygen evolution reaction (OER) and the over-reliance on scarce freshwater resources. Seawater accounts for about 96.5% of the total water reserves on Earth, providing an inexhaustible raw material for electrolysis [7,8,9,10]. Nevertheless, the high concentration of Cl^−^ in seawater leads to additional obstacles, including competitive chlorine evolution reaction (CER), electrode corrosion, and catalyst inactivation. Therefore, it is urgent to develop low-cost, high-activity and corrosion-resistant OER catalysts suitable for seawater electrolysis to realize industrial hydrogen production [11,12,13,14].

Nickel–iron layered double hydroxides (NiFe-LDHs) have attracted extensive attention as excellent non-noble metal OER catalysts due to their high intrinsic activity, adjustable chemical composition, and abundant elemental reserves [15,16,17,18,19]. The unique layered structure and synergistic interactions between Ni and Fe ions contribute to the efficient activation of water molecules and optimization of adsorption energy for reaction intermediates [20,21,22,23,24]. Nevertheless, pure NiFe-LDH suffers from poor electrical conductivity, insufficient exposed active sites, and weak corrosion resistance in Cl^−^-containing electrolytes, which seriously limit its performance under industrial working conditions [25,26,27,28]. To solve these problems, a variety of modification strategies have been developed, among which sulfur (S) doping or surface modification has been proven to be a very effective method [29,30,31]. Sulfur introduction can adjust the electronic structure of NiFe-LDH, improve charge transfer efficiency, promote the in situ generation of highly active oxyhydroxide phases, and build a protective layer to repel Cl^−^ through electrostatic repulsion, so as to enhance catalytic activity and durability [32,33].

Another important factor for practical catalyst application is the preparation method. Traditional synthetic methods of NiFe-LDH-based catalysts, such as hydrothermal and solvothermal methods, often require high temperature, long reaction times, and complex equipment, which increase production costs and are not conducive to large-scale preparation [34,35,36]. Room-temperature synthetic strategies, including electrochemical deposition, ion exchange, and spontaneous corrosion, have obvious advantages of energy saving, simple operation and good scalability, making them more suitable for industrial production [37,38,39]. For example, corrosion-driven synthesis can quickly form gradient heterostructures on porous substrates at room temperature, while electrochemical deposition can accurately control the thickness and composition of catalysts [40,41,42,43]. However, the combination of sulfur modification and room-temperature synthesis to prepare high-performance NiFe-LDH catalysts for seawater OER is still rarely reported, and the internal structure–activity relationship needs to be further clarified.

In this work, we report a simple room-temperature strategy to prepare S-modified NiFe-LDH (S-NiFe-LDH) catalysts for efficient OER and seawater electrolysis. The synthesis process combines spontaneous hydrolysis/co-precipitation and subsequent ion exchange, which can quickly form S-modified LDH nanosheets without high-temperature treatment. The introduction of S not only optimizes the electronic configuration of NiFe-LDH to enhance intrinsic catalytic activity, but also promotes the formation of a Cl^−^-repellent layer to facilitate mass transfer and corrosion resistance. The as-prepared S-NiFe-LDH catalyst shows excellent OER performance in both alkaline freshwater and seawater electrolytes, with low overpotentials and long-term stability under industrial-level current densities. This work provides a scalable and energy-efficient approach to design advanced non-noble metal catalysts for seawater splitting, and lays a foundation for sustainable hydrogen production.

## 2. Results and Discussion

### 2.1. Structural and Composition Characterization

**Figure 1:**
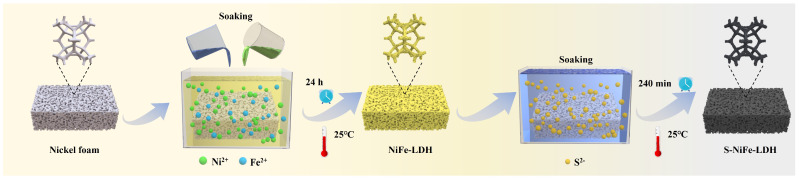
Schematic illustration of the room-temperature two-step soaking synthesis of S-NiFe-LDH catalysts on NF.

In this work, a room-temperature two-step soaking strategy was used to prepare S-modified NiFe layered double hydroxide (S-NiFe-LDH) catalyst (Figure 1). Specifically, the cleaned nickel foam (NF) was first immersed in an aqueous–isopropanol mixed solution containing nickel nitrate and ferrous sulfate at 25 °C for 24 h. The formation of NiFe-LDHs on NF follows a spontaneous corrosion–hydrolysis–co-precipitation coupled mechanism at room temperature: (1) NF acts as both the substrate and a reducing agent, undergoing mild oxidation to release Ni^2+^ and electrons; (2) Fe^2+^ in the precursor solution is oxidized to Fe^3+^ by dissolved oxygen, with electrons partially transferred from Ni^0^ in the NF; (3) the local alkalinity induced by the redox reaction and surface adsorption of OH^−^ drives the co-precipitation of Ni^2+^ (from both the precursor and NF) and Fe^3+^, forming a uniform NiFe-LDH nanosheet array in situ on the NF surface; (4) this mechanism has been verified by related literature [41,44,45]. Subsequently, the as-prepared NiFe-LDH was transferred into Na_2_S solution and soaked at 25 °C for 240 min. In this stage, S^2−^ from Na_2_S underwent ion exchange with OH^−^ and interlayer anions in NiFe-LDH, accompanied by partial surface oxidation of S^2−^ (or coordination with metal sites), thus forming S-modified NiFe-LDH on NF [46]. The possible reaction is expressed as

NiFe-LDH + zS^2−^ → NiFe-LDH-S_z_ + zOH^−^

This room-temperature synthetic route avoids high-energy conditions, and can realize large-scale preparation of S-NiFe-LDH with strong substrate adhesion and adjustable S content.

**Figure 2:**
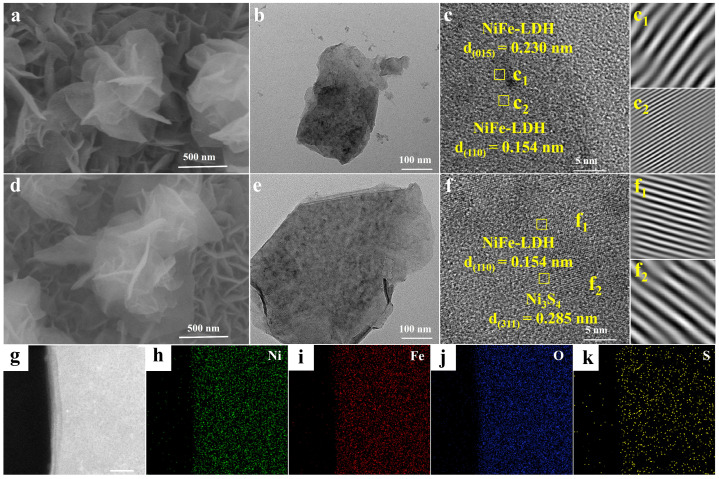
SEM image of (**a**) NiFe-LDH and (**c**) S-NiFe-LDH. Low-magnification TEM image of (**b**) NiFe-LDH and (**d**) S-NiFe-LDH. High-resolution TEM image of (**e**) NiFe-LDH and (**f**) S-NiFe-LDH. (**g**–**k**) EDX elemental mapping images of Ni, Fe, S, and O.

Scanning electron microscopy (SEM) images show that NiFe-LDH presents a typical flower-like hierarchical structure composed of interconnected ultra-thin nanosheets (Figure 2a). This open 3D structure is inherently beneficial to maximize the exposure of active sites and accelerate mass transfer in catalytic processes. Similarly, the low-magnification transmission electron microscopy (TEM) image of NiFe-LDH further confirms its thin sheet-like nanostructure (Figure 2b). To detect its crystal structure, the high-resolution TEM (HRTEM) image of NiFe-LDH shows clear lattice fringes (Figure 2c). The d-spacing of 0.230 nm corresponds to the (015) crystal plane of NiFe-LDH (inset c1), while the 0.154 nm fringe is consistent with the (110) crystal plane (inset c2), which verifies the crystal structure of the as-prepared NiFe-LDH. After vulcanization, the SEM image of S-NiFe-LDH shows that the flower-like morphology is basically preserved (Figure 2d). However, the surface of the nanosheets becomes slightly rough, which is caused by the surface vulcanization reaction between NiFe-LDH and S^2−^ in the Na_2_S solution. The low-magnification TEM image of S-NiFe-LDH confirms that the sheet-like structure is maintained (Figure 2e), indicating that vulcanization only modifies the surface without destroying the original framework of NiFe-LDH. In addition, the HRTEM image of S-NiFe-LDH shows that the 0.454 nm lattice fringe (inset f1) corresponds to the (006) crystal plane of NiFe-LDH (confirming the retention of LDH phase), while the 0.285 nm fringe (inset f2) matches the (103) crystal plane of Ni_3_S_4_ (Figure 2f). This result proves that Ni_3_S_4_ is successfully formed in situ on the surface of NiFe-LDH during vulcanization. Meanwhile, the EDX elemental mapping of S-NiFe-LDH (Figure 2g–k) shows that uniform Ni, Fe, and O signals (inherited from NiFe-LDH matrix) are observed, accompanied by uniformly dispersed S (introduced by vulcanization). This confirms that the vulcanization process proceeds uniformly on the surface of nanosheets. Furthermore, we also prepared S-NiFe-LDH catalysts with different vulcanization times (Figure S1). For S-NiFe-LDH-10, the flower-like framework is preserved, but slight surface roughness appears, corresponding to the initial adsorption of S^2−^ and local vulcanization on the LDH surface. With the further extension of catalyst vulcanization time, no obvious change is observed in the intrinsic morphology of the catalyst, but the lamellar structure of the catalyst gradually becomes thicker. At the same time, with the continuous increase in vulcanization time, the base of the catalyst begins to crack, which may be attributed to the etching effect of the S^2−^. In addition, the digital images of samples treated with different vulcanization times show that with the increase in vulcanization time (Figure S2), the color of samples changes significantly, gradually from the initial dark yellow to the final dark brown. This further indicates that the duration of vulcanization reaction has an important impact on the catalyst structure.

**Figure 3:**
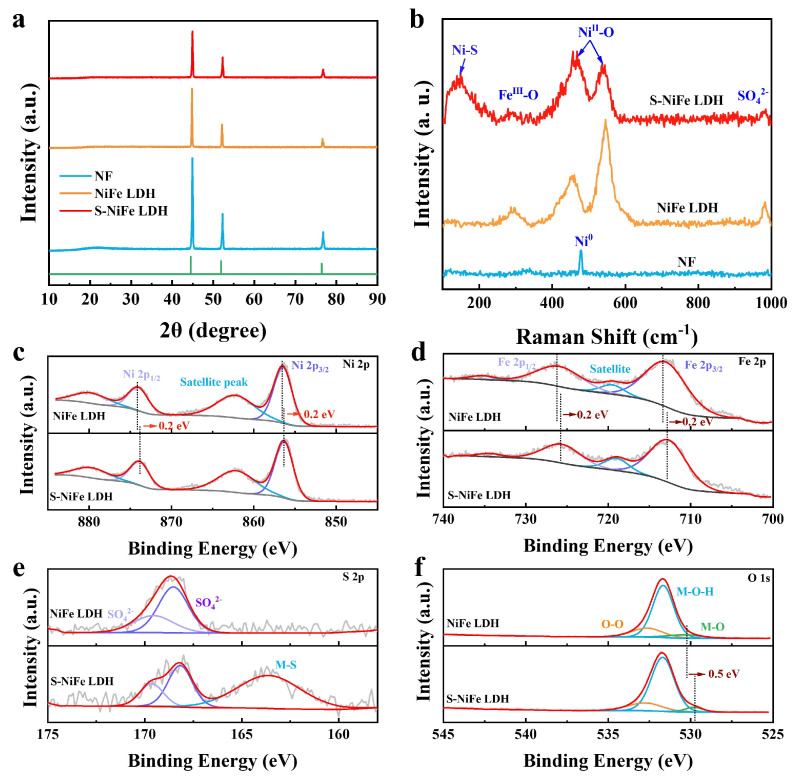
(**a**) XRD patterns and (**b**) Raman spectra of NF, NiFe-LDH and S-NiFe-LDH. XPS spectra of NiFe-LDH and S-NiFeLDH: (**c**) Ni 2p, (**d**) Fe 2p, (**e**) S 2p, and (**f**) O 1s.

To further clarify the crystal and chemical characteristics of the catalysts, X-ray diffraction (XRD) patterns were collected (Figure 3a). All samples show three obvious diffraction peaks at 44.4°, 51.8°, and 76.3°, which correspond to the Ni substrate (JCPDS 04-0850). No other obvious characteristic peaks are observed, which may be due to the relatively low crystallinity of the synthesized nanomaterials [47]. Raman spectra (Figure 3b) provide a deeper understanding of the material composition. A typical characteristic peak at 480.1 cm^−2^ appears in the NF sample, which is assigned to Ni^0^. The Ni^0^ peak is absent in NiFe-LDH and S-NiFe-LDH samples, because the nanomaterials generated during the synthesis process gradually cover the NF substrate. This result is consistent with the SEM results, where the NF substrate is completely covered by nanosheets/flowers. For the NiFe-LDH sample, two peaks at 456.4 cm^−1^ and 546.9 cm^−1^ belong to the bending vibration (*δ*_Ni-O_) and stretching vibration (*ν*_Ni-O_) of Ni-O respectively [48], while the signal centered at 291.2 cm^−1^ is attributed to Fe^III^-O. In addition, a weak peak is observed at 982.5 cm^−1^, confirming the existence of SO_4_^2−^ in NiFe-LDH sample [49]. For the S-NiFe-LDH sample, the characteristic bands of Ni-O (461.7 cm^−1^ and 539.3 cm^−1^) and Fe-O (287.5 cm^−1^) are well preserved, while a new broad peak centered at 145.7 cm^−1^ appears, which is assigned to Ni_x_S, probably resulting from the redox reactions during the etching process [50,51]. Meanwhile, we find that the intensity ratio of *δ*_Ni-O_ peak to *ν*_Ni-O_ peak decreases gradually with the extension of vulcanization time (Figure S3). This may be due to the breaking of Ni-O bonds during vulcanization, the destruction of layered structure, and the transformation of Ni from hydroxide phase to sulfide phase. Alternatively, the electronegativity of S^2−^ is lower than that of O^2−^, and its adsorption or doping will change the electron cloud distribution around Ni atoms, resulting in a decrease in bond energy and an increase in bond length of Ni-O bonds [52,53].

X-ray photoelectron spectroscopy (XPS) was used to detect the surface chemical states and electronic structure of NiFe-LDH and S-NiFe-LDH. The full spectrum (Figure S4) confirms the existence of Ni, Fe, O, and S, which is consistent with TEM-EDS mapping (Figure 2g–k). The S element in NiFe-LDH comes from SO_4_^2−^, which has been confirmed by the previous Raman spectrum results. For the Ni 2p XPS spectrum (Figure 3c), two prominent characteristic peaks centered at 856.4 and 874.1 eV can be clearly seen in the NiFe-LDH sample, which directly prove the existence of Ni 2p_3/2_ and Ni 2p_1/2_ species in the sample. At the same time, the satellite peaks related to these Ni 2p species are detected at 862.3 and 880.1 eV respectively, which is consistent with the typical spectral characteristics of Ni-containing LDH [54,55]. For the Fe 2p spectrum (Figure 3d), the main peaks at 713.3 and 726.2 eV can be clearly attributed to Fe 2p_3/2_ and Fe 2p_1/2_ orbitals, confirming that Fe species are successfully introduced into the LDH framework [56,57]. Notably, after S is introduced into the NiFe-LDH matrix by doping treatment, the Ni 2p and Fe 2p peaks show a slight negative shift of about 0.2 eV compared with the undoped NiFe-LDH. As shown in the S 2p spectrum (Figure 3e), a distinct peak appearing at 163.6 eV is the characteristic of metal–sulfur (M-S) bonds, verifying the formation of chemical interactions between S and the metal centers (Ni/Fe) in the doped sample [58,59]. In addition, two weak peaks at 168.1 eV and 169.6 eV are also detected, which are attributed to the intercalated SO_4_^2−^ in the LDH interlayer, a common by-product in the synthesis of LDH materials [49,60]. Furthermore, the O 1s spectrum (Figure 3f) can be divided into three well-resolved components, corresponding to metal–oxygen (M-O) bonds (529.7 eV), metal–hydroxyl (M-OH) groups (531.7 eV), and surface-adsorbed water (O-O) molecules (532.6 eV) respectively [61]. Compared with pure NiFe-LDH, the M-O bond-related peak in S-NiFe-LDH shows a significant positive shift (about 0.5 eV). In conclusion, these changes in binding energies of Ni 2p, Fe 2p, O 1s, and S 2p core levels strongly indicate that S doping has significantly modified the local electronic environment of Ni and Fe centers in the LDH structure [62].

### 2.2. OER Performance Test

**Figure 4:**
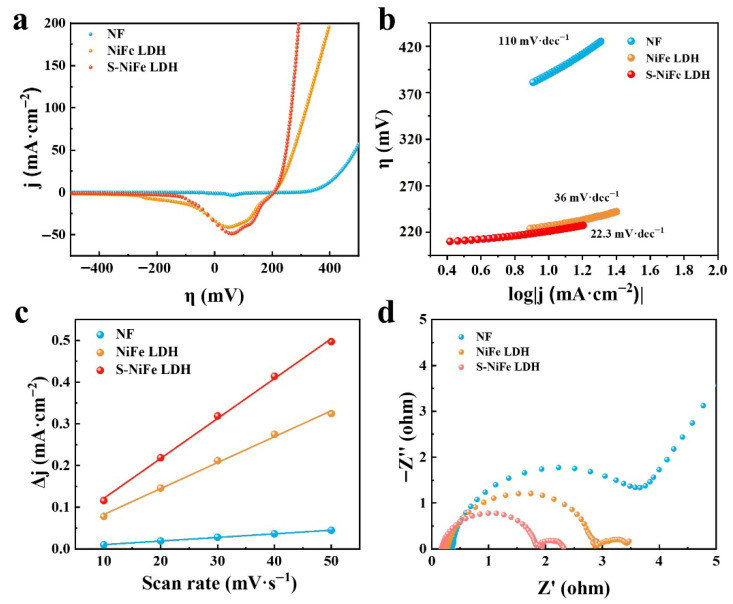
(**a**) LSV curves, (**b**) Tafel slopes, (**c**) linear fitting of double-layer capacitance, and (**d**) EIS Nyquist plots of NF, NiFe-LDH, and S-NiFe-LDH samples.

The electrocatalytic performance was evaluated by a standard three-electrode system in 1.0 M KOH electrolyte, and linear sweep voltammetry (LSV) tests were carried out under 90% iR compensation to characterize OER activity. As shown in Figure 4a, the reduction peak of all samples around 1.28 V vs. RHE comes from the transformation of Ni^3+^ to Ni^2+^ [63]. S-NiFe-LDH shows significantly enhanced OER activity compared with bare NF and pure NiFe-LDH, reflected in the faster rise in current density at lower applied potential. To quantify this activity advantage, Figure S5 compares the reaction potentials of NiFe-LDH and S-NiFe-LDH at different current densities. At 50 mA·cm^−2^, pure NiFe-LDH needs an overpotential of 280 mV, while S-NiFe-LDH can reach the same current density at a reduced potential of 250 mV, confirming the activating effect of S introduction. This performance difference remains with the increase in current density: at 100 mA·cm^−2^, NiFe-LDH shows an overpotential of 320 mV, while S-NiFe-LDH maintains a lower overpotential of 270 mV. Even at a high current density of 200 mA·cm^−2^, S-NiFe-LDH still maintains its activity superiority (290 mV vs. 400 mV for NiFe-LDH), surpassing many previously reported OER electrocatalysts (Tables S1 and S2) and highlighting its excellent catalytic performance. The Tafel slope, a key parameter describing OER kinetics, was derived from LSV curves (Figure 4b). S-NiFe-LDH has a much smaller Tafel slope (22.3 mV·dec^−1^) than NiFe-LDH (36 mV·dec^−1^) and NF (110 mV·dec^−1^), indicating that S doping accelerates OER kinetics. We also studied the OER performance of the S-NiFe-LDH catalysts prepared at different reaction times, as shown in Figure S6. It was found that OER activity increases with the prolongation of reaction time, and the S-NiFe-LDH-240 sample has the best performance. The electrochemically active surface area (ECSA), which is related to the density of active sites, was evaluated by electrochemical double-layer capacitance (C_dl_) measurement [64]. By plotting the capacitive current (Δ_j_/2) against the scan rate (Figure 4c and Figure S7), the S-NiFe-LDH sample shows a *C*_dl_ value of 9.57 mF·cm^−2^, which is 1.54 times that of NiFe-LDH catalyst and more than six times that of NF substrate, indicating that the S-doped catalyst has more active sites. Electron transfer kinetics was further studied by electrochemical impedance spectroscopy (EIS). As shown in Figure 4d, the S-NiFe-LDH sample has a smaller semicircle diameter in the Nyquist plot than NiFe-LDH, indicating a faster charge-transfer rate and more favorable OER dynamics.

Although direct quantitative elemental analysis of sulfur loading was not conducted in this work, the degree of sulfur incorporation and surface reconstruction across the S-NiFe-LDH-t series can be effectively modulated by precisely controlling the sulfidation duration. With the extension of sulfidation time, the gradual enhancement of characteristic S-related signals in XPS and Raman spectra clearly verifies the progressive increase in sulfur species on the catalyst surface. Meanwhile, the OER catalytic activity exhibits a continuous and regular evolution trend that is well correlated with the sulfidation degree regulated by reaction time. Such consistent qualitative variation enables a reasonable structure–activity relationship to be established between sulfur incorporation level and electrochemical performance.

### 2.3. OER Performance in Alkaline Seawater

**Figure 5:**
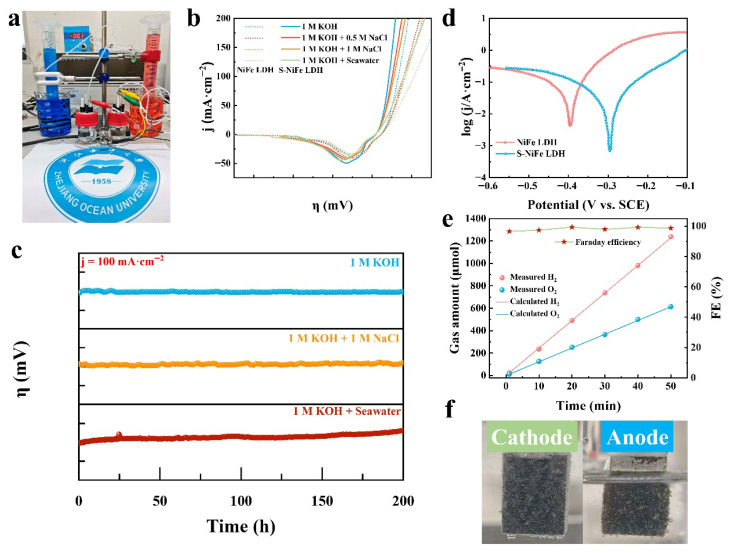
(**a**) Digital images of three-electrode electrolysis of water in an H-type electrolytic cell. (**b**) LSV curves of S-NiFe-LDH electrocatalysts under different electrolytes. (**c**) I-t curves of S-NiFe-LDH in 1 M KOH, 1 M KOH + 1 M NaCl, and 1 M KOH + seawater, respectively. (**d**) Corrosion polarization curve of NiFe-LDH and S-NiFe-LDH. (**e**) Theoretical and measured gaseous products (H_2_ and O_2_) from S-NiFe-LDH at 100 mA cm^−2^ in 1 M KOH + seawater electrolyte. (**f**) Photograph of the cathode (S-NiFe-LDH) and anode (graphite flake) at a current density of 100 mA·cm^−2^ in 1 M KOH + seawater electrolyte.

Benefiting from the promising OER activity of S-NiFe-LDH observed in previous tests, we further extended the performance evaluations to practical electrolyte systems. Specifically, we tested three systems: alkaline simulated seawater (1 M KOH + 0.5 M NaCl, 1 M KOH + 1 M NaCl) and alkaline natural seawater (1 M KOH + seawater). To fully characterize the catalyst performance in these media, we used a self-built gas collection device to simultaneously quantify the gas products generated during electrolysis (Figure 5a). As shown in Figure 5b, S-NiFe-LDH needs overpotentials of 320 mV and 340 mV to reach a current density of 200 mA cm^−2^ in 1 M KOH + 0.5 M NaCl and 1 M KOH + 1 M NaCl respectively. These values are very close to its performance in pure 1 M KOH electrolyte. Compared with the pure KOH system, the performance decline in alkaline seawater is mainly attributed to the formation of insoluble precipitates blocking the electrode surface, which is consistent with previous reports on electrocatalysts for seawater electrolysis [65,66]. Stability is a key parameter to evaluate the practical application of catalysts, especially under the harsh conditions of seawater electrolysis. To evaluate this, we carried out long-term stability tests at a constant current density of 100 mA·cm^−2^ in all three electrolytes. As shown in Figure 5c, after 200 h of continuous operation in alkaline seawater, the current density only decreases slightly (retaining about 88.7% of the initial value), which is slightly lower than the retention rate in 1 M KOH (97.3%). This result confirms that S-NiFe-LDH has excellent electrocatalytic activity and stability in different electrolyte systems. In addition, the corrosion polarization curves (Figure 5d) show that S-NiFe-LDH has a more positive corrosion potential than pure NiFe-LDH in 1 M KOH + seawater. A more positive corrosion potential means lower corrosion tendency, proving that sulfur modification effectively improves the corrosion resistance of NiFe-LDH [67,68]. We also measured the Faradaic efficiency (FE) of the electrolyzer based on S-NiFe-LDH at a high current density of 100 mA·cm^−2^ by collecting and quantifying gas products using the water displacement method (Figure 5a). As shown in Figure 5e, the measured molar ratio of H_2_ to O_2_ is very close to the theoretical 2:1 stoichiometry of water splitting. The OER and HER FEs of S-NiFe-LDH in seawater electrolyte are both close to 100%, meaning almost all electrons are used for water electrolysis. This result further verifies the excellent Cl^−^ corrosion resistance of the catalyst. Finally, the in situ photos of electrode surfaces during electrolysis (Figure 5f) show gas evolution behavior consistent with previous observations. At the S-NiFe-LDH anode, large O_2_ bubbles form and adhere to the surface, which may be due to the low solubility of O_2_ in water, leading to rapid accumulation on the electrode and intermolecular forces promoting bubble coalescence. In contrast, the graphite cathode shows a dense distribution of small H_2_ bubbles. This difference is because H_2_ has extremely low solubility in water, allowing it to diffuse quickly from the cathode surface; this rapid diffusion limits bubble merging, thus maintaining small bubble sizes.

**Figure 6:**
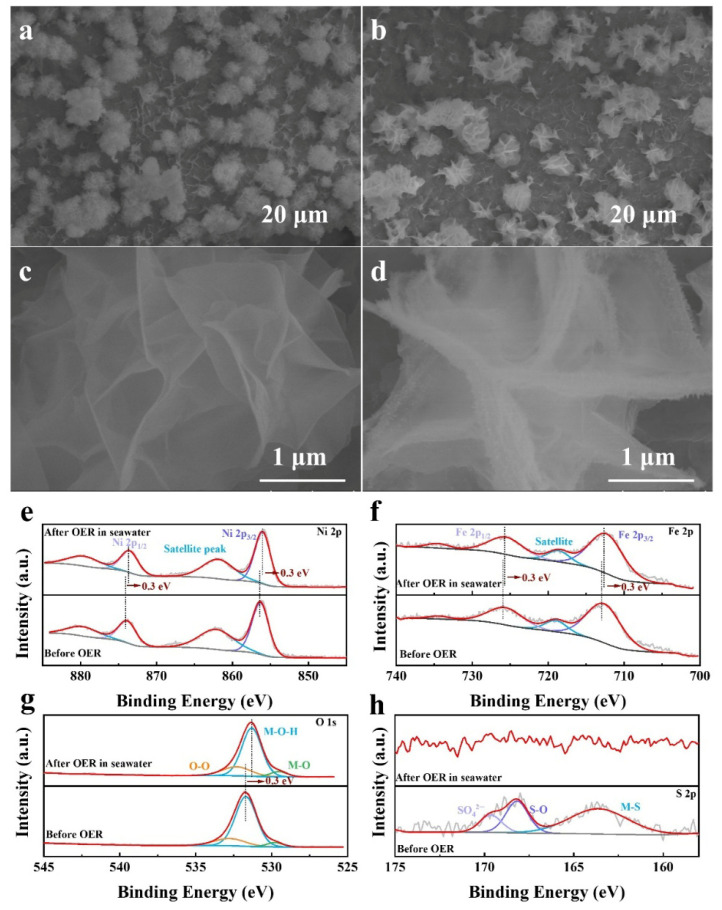
SEM images of S-NiFe-LDH (**a**,**c**) before and (**b**,**d**) after OER stability reactions. XPS spectrum of S-NiFe-LDH before and after OER stability reactions: (**e**) Ni 2p; (**f**) Fe 2p; (**g**) O 1 s; (**h**) S 2p.

To further understand the structural and chemical stability of S-NiFe-LDH under the harsh conditions of seawater electrolysis, we used SEM and XPS to characterize the catalyst before and after 200 h of OER operation. As shown in the initial SEM images (Figure 6a,c), the as-prepared catalyst has a uniformly distributed porous nanosheet array structure. After 200 h of continuous OER in seawater, the nanosheet morphology is basically intact (Figure 6b,d), without obvious particle agglomeration or structural collapse. Such stable morphological features directly prove the robust structural stability of S-NiFe-LDH, even under the long-term corrosive environment of seawater electrolysis. Subsequent XPS tests were carried out to clarify the evolution of the catalyst’s chemical state during OER, which is crucial for understanding the dynamic behavior of active sites. After 200 h of testing, the subtle differences between XPS full spectra (Figure S8) further confirm the chemical changes of S-NiFe-LDH after OER. Focusing on the high-resolution spectra, the Ni 2p spectrum (Figure 6e) shows an obvious positive binding energy shift after the reaction; a similar trend is also observed in the Fe 2p spectrum (Figure 6f). These shifts indicate that during the anodic OER process, electron rearrangement occurs on the catalyst surface, which can optimize the adsorption energy of reaction intermediates and thus accelerate OER kinetics [46,69]. In the O 1s spectra (Figure 6g), a slight negative shift in binding energy is detected after the stability test, implying the formation of more metal hydroxy oxides on the catalyst surface [70]. For the S 2p spectrum (Figure 6h), partial dissolution of surface sulfur species is observed after OER. Notably, this dissolution process is accompanied by the oxidation of residual sulfur, which drives the in situ surface reconstruction of the catalyst [71]. This reconstruction leads to the generation of unsaturated active sites, such as metal vacancies or coordinatively unsaturated metal centers. Importantly, this not only enhances the intrinsic catalytic activity but also strengthens the electrode stability by dynamically supplementing the active sites that may be consumed during the reaction. Taken together, these characterization results demonstrate that S-NiFe-LDH undergoes coupled surface reconstruction and valence state transitions during seawater OER, which together support its excellent stability and catalytic performance under harsh electrolytic conditions. Furthermore, the S-NiFe-LDH nanosheet arrays are in situ-grown on the nickel foam substrate via strong chemical interaction rather than physical coating, which affords robust mechanical stability and outstanding adhesion. During the long-term seawater electrolysis with intensive gas bubbling, the catalyst film shows no obvious peeling, cracking or detachment. After the 200 h stability test, the hierarchical nanosheet morphology is well preserved without structural degradation or separation from the substrate, confirming the excellent mechanical robustness of the as-prepared catalyst.

### 2.4. Theoretical Calculations

**Figure 7:**
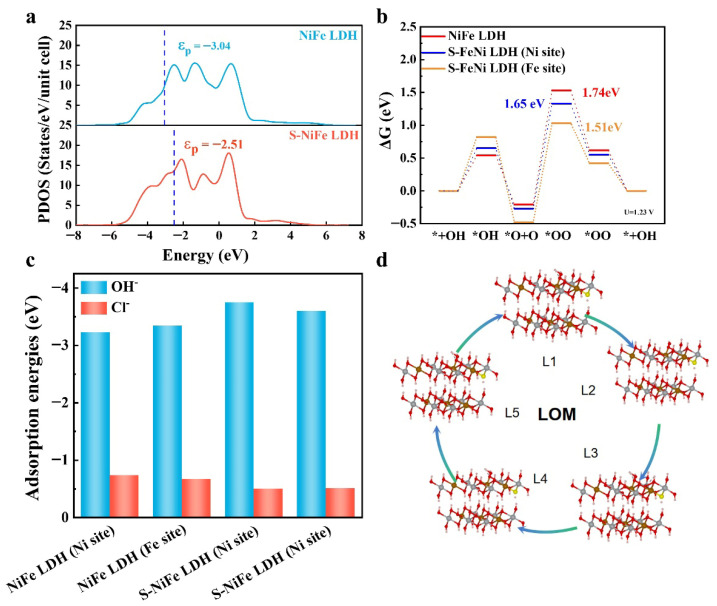
DFT insights into the enhanced seawater OER activity of S–NiFe-LDH via the lattice oxygen mechanism. (**a**) PDOS of O 2p orbitals for NiFe-LDH and S-NiFe-LDH, showing the upshifted O p-band center upon S doping. (**b**) Calculated free-energy profiles of the oxygen evolution reaction (OER) via the lattice oxygen mechanism (LOM) on NiFe-LDH (111) and S–NiFe-LDH (111) surfaces at the equilibrium potential (U = 1.23 V vs. RHE). (**c**) Calculated adsorption energies of OH^−^ and Cl^−^ on NiFe-LDH (111) and S-NiFe-LDH (111) surfaces, demonstrating the selective adsorption behavior induced by sulfur doping. (**d**) Schematic illustration of the lattice oxygen mechanism (LOM)-mediated OER process on S-NiFe-LDH, highlighting the dynamic lattice oxygen activation and catalytic cycle.

To reveal the fundamental reason for the enhanced OER performance and seawater stability of S-doped NiFe-LDH, we carried out a series of density functional theory (DFT) calculations to systematically study the electronic structure, reaction thermodynamics, ion adsorption behavior and catalytic mechanism. The projected density of states (PDOS) of O 2p orbitals was calculated to clarify the electronic regulation caused by sulfur incorporation (Figure 7a). For pure NiFe-LDH, the O 2p-band center is located at −3.04 eV relative to the Fermi level. After sulfur doping, the O 2p-band center of S-NiFe-LDH is significantly upshifted to −2.51 eV, much closer to the Fermi level. This upshift is a direct result of the enhanced orbital hybridization between O 2p and metal (Ni/Fe) 3d orbitals, which strengthens the covalency of Ni/Fe-O bonds. Importantly, the increased O p-band center is a recognized descriptor for lattice oxygen activation, indicating a significantly higher tendency of lattice oxygen to participate in O-O coupling reactions. This electronic regulation lays a foundation for the transition of OER pathway from the traditional adsorbate evolution mechanism (AEM) to the lattice oxygen mechanism (LOM), which is known to break through the thermodynamic limitations of AEM and accelerate reaction kinetics. Based on the electronic structure insights, we further calculated the free-energy profiles of OER pathway on NiFe-LDH and S–NiFe-LDH surfaces at equilibrium potential (U = 1.23 V, Figure 7b). For pure NiFe-LDH, the potential-determining step (PDS) corresponds to the O-O coupling step (*O + O_lattice → *OO) in the LOM pathway, with a high thermodynamic barrier of 1.74 eV. In contrast, sulfur modification greatly reduces this energy barrier: the PDS barriers of S-NiFe-LDH at Ni site and Fe site are only 1.65 eV and 1.51 eV, respectively. The significantly reduced energy barrier confirms that sulfur doping effectively stabilizes the key oxygen intermediates involved in O-O bond formation, directly overcoming the thermodynamic limitation of the rate-determining step and accelerating the overall OER kinetics. This result is in good agreement with the upshifted O 2p-band center observed in PDOS analysis, because the activated lattice oxygen promotes the O-O coupling process in the LOM pathway. To clarify the excellent corrosion resistance and operational stability of S-NiFe-LDH in alkaline seawater, we calculated the adsorption energies of OH^−^ (the OER reactant) and Cl^−^ (main competitive ion in seawater) on the catalyst surfaces (Figure 7c). Compared with pure NiFe-LDH, sulfur incorporation significantly enhances the adsorption of OH^−^, which is essential for initiating the OER process and maintaining the catalytic cycle. At the same time, the adsorption of Cl^−^ on S-NiFe-LDH is significantly weakened. This selective adsorption behavior is crucial: it not only promotes OER kinetics by ensuring sufficient OH^−^ supply to active sites, but also effectively inhibits the competitive chlorine evolution reaction (CER) and prevents Cl^−^-induced catalyst corrosion. This dual effect provides a clear theoretical explanation for the superior long-term stability of S-NiFe-LDH in actual seawater electrolysis. Based on the above electronic structure, thermodynamic and adsorption calculations, we propose a complete LOM-mediated OER mechanism for S-NiFe-LDH (Figure 7d). The catalytic cycle starts with the selective adsorption of OH^−^ on the catalyst surface to form *OH intermediates, which then undergo deprotonation to generate surface *O species. Benefiting from the sulfur-induced lattice oxygen activation (evidenced by the upshifted O 2p-band center), surface *O species easily couple with adjacent lattice oxygen to form O-O bonds, releasing molecular O_2_ and generating transient oxygen vacancies. The oxygen vacancies are then supplemented by OH^−^ from the electrolyte, restoring the lattice oxygen framework and completing the catalytic cycle. Sulfur doping plays a dual role in this process. It promotes lattice oxygen activation to facilitate LOM, and stabilizes the dynamic oxygen redox process during the cycle, thus simultaneously enhancing the catalytic activity and long-term durability of S–NiFe-LDH under harsh seawater electrolysis conditions.

## 3. Conclusions

In summary, we have successfully developed a simple room-temperature two-step soaking method to prepare S-modified NiFe-LDH catalysts for efficient and stable OER in alkaline fresh water and seawater electrolytes. The synthetic strategy avoids high-temperature and complex equipment, enabling large-scale production with strong substrate adhesion and adjustable sulfur content. The introduction of sulfur into NiFe-LDH brings multiple synergistic advantages: (1) optimizing the electronic structure of Ni and Fe active sites, thus enhancing intrinsic catalytic activity and accelerated OER kinetics; (2) inducing the formation of a Cl^−^-repellent layer and improving corrosion resistance, effectively alleviating electrode degradation in Cl^−^-rich seawater; and (3) increasing the electrochemical active surface area and promoting mass transfer. Collectively, our theoretical and mechanistic insights show that sulfur doping endows NiFe-LDH with favorable electronic modulation, accelerated LOM-OER kinetics and excellent Cl^−^ corrosion resistance, establishing a promising platform for large-scale alkaline seawater electrolysis.

## Data Availability

The original contributions presented in this study are included in the article. Further inquiries can be directed to the corresponding authors.

## Supplementary Materials

The following supporting information can be downloaded at https://www.mdpi.com/article/10.3390/mi17060675/s1. Figure S1. The SEM images of (a,b) NiFe-LDH; (k,l) S-NiFe-LDH-10; (c,d) S-NiFe-LDH-30; (e,f) S-NiFe-LDH-60; (g,h) S-NiFe-LDH-120 and (i,j) S-NiFe-LDH-240. Figure S2. The digital photograph of time-dependent color evolution of NiFe-LDH during sulfur modification in Na_2_S solution. Figure S3. Raman spectra of NiFe-LDH and S-NiFe-LDH-t. Figure S4. XPS survey spectrum of NiFe-LDH and S-NiFe-LDH. Figure S5. The corresponding overpotentials at 50, 100, and 200 mA·cm^−2^ of NF, NiFe-LDH, and S-NiFe-LDH. Figure S6. LSV curves of S-NiFe-LDH-t samples. Figure S7. CV curves of (a) NF, (b) NiFe-LDH, and (f) S-NiFe-LDH in a nonfaradaic region with scan rate of 10–50 mV s^−1^. Figure S8. XPS survey spectrum of S-NiFe-LDH before and after OER stability reactions. Table S1. Comparison of Ni-based catalyst reported recently for OER electrocatalytic performance. Table S2. Summary of electrocatalytic performance for OER catalysts in seawater. References [72,73,74,75,76,77,78,79,80,81,82,83,84,85,86,87,88,89,90,91,92,93] are cited in the supplementary materials.
