# Supplementary material for: Controlled-Atmosphere Corrosion Engineering Toward NiFe-LDH Enabling High-Performance Alkaline Seawater Electrolysis with Long-Term Stability
Source: Micromachines (Basel). 2026 May 29;17(6):675. doi: 10.3390/mi17060675 (PMC13303408; doi:10.3390/mi17060675)
Supplement: Supplementary file 1 [file micromachines-17-00675-s001.zip › micromachines-4273812-supplementary.pdf]

micromachines

MDPI

Supplementary Materials

Controlled-Atmosphere Corrosion Engineering Toward NiFe-LDH Enabling High-Performance Alkaline Seawater Electrolysis with Long-Term Stability

Yang Su [1,2], Yuqing Li [1], Qing Wang [1], Yue Hu [1], Liu Han [3], Xiyuan Feng [4,*], Bin Wu [5,6], Jie Wang [3] and Yingtang Zhou [1,2,*]

[1] Zhejiang Key Laboratory of Pollution Control for Port–Petrochemical Industry, Marine Science and Technology College, Zhejiang Ocean University, Zhoushan 316022, China; suyang@zjou.edu.cn (Y.S.); liyuqing@zjou.edu.cn (Y.L.); wq1398903367@163.com (Q.W.); huyue0214@zjou.edu.cn (Y.H.)
[2] National Engineering Research Center for Marine Aquaculture, Zhejiang Ocean University, Zhoushan 316022, China
[3] School of Chemistry and Chemical Engineering, Anhui Provincial Key Laboratory of Advanced Catalysis and Energy Materials, Anhui Key Laboratory of Optoelectronic Magnetic Functional Complex and Nano Complex, Anqing Normal University, Anqing 246011, China; hanliu@aqnu.edu.cn (L.H.); wangjie@aqnu.edu.cn (J.W.)
[4] School of Integrated Circuits (School of Microelectronics), Northwestern Polytechnical University, Xi'an 710129, China
[5] School of Materials Science and Engineering, Nanyang Technological University, Singapore 639798, Singapore; bin.wu@ntu.edu.sg
[6] Hubei Key Laboratory of Plasma Chemistry and Advanced Materials, Wuhan Institute of Technology, Wuhan 430205, China
* Correspondence: fengxy@nwpu.edu.cn (X.F.); zhouyingtang@zjou.edu.cn (Y.Z.)

1. Experimental Section

1.1. Materials and Reagents

All chemicals were used as received without further purification. Nickel nitrate hexahydrate ($Ni(NO_3)_2{\cdot}6H_2O$, 99%), Iron sulfate heptahydrate ($FeSO_4{\cdot}7H_2O$, >99 %), sodium sulfide nonahydrate ($Na_2S{\cdot}9H_2O$, 98%), potassium hydroxide (KOH, 85%), sodium chloride (NaCl, 99.5%), hydrochloric acid (HCl, 37%), acetone (99.5%), and ethanol (99.7%) were purchased from Aladdin Reagent Co., Ltd. Nickel foam (NF, thickness: 1 mm, porosity: ~95%, PPI: 110) was obtained from Kunshan Guangshengjia New Materials Co., Ltd. Deionized water (DI water, resistivity ≥ 18.2 MΩ·cm) was used for all solution preparations.

1.2. Pretreatment of Nickel Foam (NF)

The NF was first cut into rectangular pieces with dimensions of 1 cm × 2 cm and sequentially ultrasonicated in 1 M HCl, acetone, ethanol, and deionized DI water for 15 min each to thoroughly remove surface oxides, organic contaminants, and other impurities. The pretreated NF was then dried in a vacuum oven at 60 °C for 20 min to remove moisture and stored in a desiccator to prevent reoxidation prior to subsequent synthesis.

1.3. Room-Temperature Synthesis of NiFe-LDH

NiFe-LDH were fabricated with slight modifications based on a previously reported work [1,2]. Specifically, 1.78 g of $Ni(NO_3)_2{\cdot}6H_2O$ and 0.24 g of $FeSO_4{\cdot}7H_2O$ were separately dissolved in 30 mL of EtOH and 10 mL of DW, respectively. The two precursor solutions were then combined under intense magnetic stirring to yield a green turbid mixture. A pre-cleaned NF substrate was fully submerged in the resulting mixed solution at 25 °C for

24 h. Subsequent to the reaction, the sample was rinsed thoroughly with DW and dried in a vacuum oven at 50 °C for 6 h to obtain the target NiFe-LDH catalyst.

1.4. Room-Temperature Synthesis of S-NiFe-LDH-t (t = 10, 30, 60, 120, 240)

Sulfur modification was achieved via in situ ion exchange at room temperature to avoid high-temperature-induced structural collapse of NiFe-LDH. First, 20 mL of 0.1 M $Na_2S{\cdot}9H_2O$ aqueous solution were prepare, and stirred magnetically at 25 °C for 15 min to ensure complete dissolution. Then the as-prepared NiFe-LDH were immersed into the above Na_2S solution and maintained a static reaction at 25 °C for desired time, during which S^{2-} ions undergo ion exchange with interlayer anions (SO_4^{2-}) and surface OH^- of NiFe-LDH along with coordination with Ni/Fe active sites. Finally, the modified electrode was rinsed thoroughly with DI water and ethanol to remove physically adsorbed S species and residual Na^+ ions, and dry it in a vacuum oven at 60 °C for 5 h to obtain the target S-NiFe-LDH catalyst.

1.5. Physical Characterization

The crystal phase composition of the prepared materials was analyzed using the D/MAX-2500/PC X-ray diffraction (XRD) instrument. The morphology of the samples was examined using a Hitachi S4800II scanning electron microscope (SEM) and an Titan ETEM transmission electron microscope (TEM). The Thermo ESCALAB 250XI X-ray photoelectron spectroscopy (XPS) instrument was used to analyze the electronic structure and chemical composition of the sample surfaces. The LabRamHR800 Raman spectrometer, operating at a wavelength of 532 nm, was used for in-situ Raman analysis.

Notes: The XRD patterns of all samples (nickel foam, NiFe-LDH/nickel foam, and S-modified NiFe-LDH/nickel foam) were recorded directly on the as-prepared samples without any modification treatments such as grinding, peeling, or powdering, which ensures the authenticity of the surface phase information. Each sample was cut into a 10 mm × 10 mm piece and gently attached to the sample stage with conductive adhesive to keep the test surface flat and avoid signal distortion caused by sample tilting. The measurements were performed using a standard powder diffractometer in glancing incidence mode (incidence angle = 0.5°), which effectively focuses on the surface catalyst layer and minimizes the interference from the nickel foam substrate. Although the inherent porous structure and surface roughness of nickel foam lead to slight baseline fluctuations and minor peak broadening, these phenomena do not interfere with the identification of characteristic diffraction peaks. Specifically, confocal microscopic Raman mode was applied to precisely focus the laser beam on flat and homogeneous regions of the catalyst surface, avoiding rough and protruding sites of the nickel foam substrate. For each sample, at least three random microdomains were measured, and the final spectrum was obtained by multi-point averaging to reduce random error induced by local surface undulation. A suitably low laser power was adopted throughout the test to prevent sample thermal damage and baseline distortion. With these standardized testing strategies, the interference originating from substrate surface irregularities was effectively minimized, guaranteeing the accuracy and reproducibility of Raman spectral analysis.

1.6. Electrochemical Measurements

All electrochemical measurements were performed on a CHI 760E electrochemical workstation using a three-electrode system at room temperature. The as-prepared products with an effective geometric area of 1 × 2 cm^2 were employed as working electrodes, with a Hg/HgO electrode and a graphite rod serving as the reference and counter electrodes, respectively. Three types of alkaline electrolytes (pH = 14) were utilized, namely 1 M KOH, 1 M KOH + 0.5 M NaCl, and 1 M KOH + seawater. The potentials measured in

https://doi.org/10.3390/xxxxx

the 1 M KOH electrolyte were converted to the reversible hydrogen electrode (RHE) scale using Equation (S1):

$$E\ (RHE) = E\ (Hg/HgO) + 0.098 + 0.0591 \times pH \quad (S1)$$

The overpotential (η) for the oxygen evolution reaction was calculated according to the following equation (S2):

$$\eta = E\ (V\ vs.\ RHE) - 1.23\ V \quad (S2)$$

Linear sweep voltammetry (LSV) measurements for the OER were conducted at a scan rate of 5 $mV·s^{-1}$, and the obtained curves were corrected by manual 90% iR-compensation based on the corresponding uncompensated solution resistance R_s. Specifically, the selection criteria for Tafel slope calculation, including the specific fitting range (focusing on the low-overpotential kinetic control region, which avoids the interference of redox peak regions and mass-transport limitation regions), the judgment standard for the linear segment (requiring the correlation coefficient ($R^2 > 0.999$) to ensure the reliability of the linear fitting), and the exclusion rules for interference regions (excluding the redox peak region where the reaction is not dominated by kinetic control and the mass-transport limitation region where the test results are easily distorted). Meanwhile, we have explicitly specified that the same standardized fitting range and calculation method are consistently applied to all catalyst samples, which effectively ensures the comparability of Tafel slope data between different samples and fully addresses the rationality concern of Tafel slope calculation.

Electrochemical impedance spectroscopy (EIS) tests were performed at a constant current density of 10 $mA·cm^{-2}$ in 1 M KOH electrolyte, with an applied amplitude of 5 mV and a frequency range spanning from 10^5 to 10^{-1} Hz. Chronoamperometric (i-t) curves were recorded to evaluate the long-term electrochemical durability of the catalysts across the aforementioned electrolytes. To determine the double-layer capacitance C_{dl} without involving faradaic reactions, cyclic voltammetry (CV) measurements were carried out in a non-faradaic potential window, with scan rates varying from 10 to 50 $mV·s^{-1}$. The electrochemical active surface area (ECSA) was further calculated according to Equation (S3):

$$ECSA = S \times C_{dl}/C_s \quad (S3)$$

where S denotes the effective geometric area of the electrode, and C_s represents the C_{dl} value of the smooth nickel foam framework substrate.

The faradaic efficiency (FE) of the overall water spilling was quantified via the drainage method in a sealed H-type electrolytic cell. It was calculated as the ratio of the experimentally measured H_2/O_2 yields to the theoretically calculated amounts, following Equation (S4):

$$FE\ (\%) = n·m_2·F/I·t \times 100\% \quad (S4)$$

Herein, n refers to the number of electron transfers (2 for the hydrogen evolution reaction (HER) and 4 for the OER), m_2 is the molar amount of the collected gaseous products (H_2 or O_2), F is the Faraday constant (96500 C mol^{-1}), I is the applied current, and t is the electrolysis time.

The natural seawater adopted in this work was collected from coastal surface water. Before the test, raw seawater was subjected to natural standing sedimentation and membrane filtration to remove suspended solids and particulate contaminants. The obtained treated natural seawater exhibits typical near-neutral pH and high conductivity characteristics consistent with common coastal seawater, containing dominant ions such as Cl^-, Na^+, Mg^{2+}, Ca^{2+} and SO_4^{2-} as well as trace natural organic impurities. All OER measurements were conducted directly using the pre-treated natural seawater without further chemical adjustment.

https://doi.org/10.3390/xxxxx

2. Calculation Methods and Details

All density functional theory (DFT) calculations were performed via the *Vienna ab* initio simulation package (VASP) based on MedeA software, using the generalized gradient approximation (GGA)-Perdew–Burke–Ernzerhof(PBE) exchange correlation functional and projector augmented wave (PAW) pseudopotential [3]. A kinetic energy cutoff of 500 eV was set for plane-wave basis sets[4].

According to our previous literature[5], FeNi layered double hydroxide (FeNi LDH) was used as the base model with lattice parameters a = 9.28400 Å, b = 12.03000 Å , c = 25.00000 Å, α = β = 90.0000, γ = 115.4579. S-doped FeNi LDH (S-FeNi LDH) was constructed by substituting lattice oxygen (O) with sulfur (S) in FeNi LDH, followed by full lattice optimization for the doped system. A 15 Å vertical vacuum layer was introduced to eliminate spurious interactions between adjacent periodic images. A 1 × 5 × 1 Monkhorst–Pack k-point mesh was applied for Brillouin zone sampling of both bulk and surface models.

Electronic convergence was set to 10^{-5} eV, and ionic relaxation converged until the maximum residual force on each atom was less than Å. DFT + U corrections (2.5 eV for Fe and Ni 3d orbitals) were adopted to describe strong on-site Coulomb interactions [4]. The p-band center (Ep) of surface heteroatoms (O/S) was calculated as the first moment of the p-projected density of states (p-PDOS) below the Fermi level (EF) according to the standard formula [5]:

$$E_p = \frac{\int_{-\infty}^{E_F} E \cdot PDOS(E)dE}{\int_{-\infty}^{E_F} PDOS\,(E)dE}$$

The adsorption energy (ΔEads) was defined as Equation (S5):

$$\Delta E_{ads}=E_{ad/sub}-E_{ad}-E_{sub} \quad \text{(S5)}$$

where $E_{ad/sub}$,E_{ad}, and E_{sub} represent the total energy of the optimized adsorbate-substrate system, the isolated adsorbate, and the clean catalyst surface (FeNi LDH or S-FeNi LDH), respectively.

The Gibbs free energy change (ΔG) at 300 K was calculated as Equation (S6):

$$\Delta G = \Delta E + \Delta ZPE - T\Delta S \quad \text{(S6)}$$

where ΔE is the DFT-calculated total energy difference, ΔZPE is the zero-point energy difference, and $T\Delta S$ is the entropic contribution.

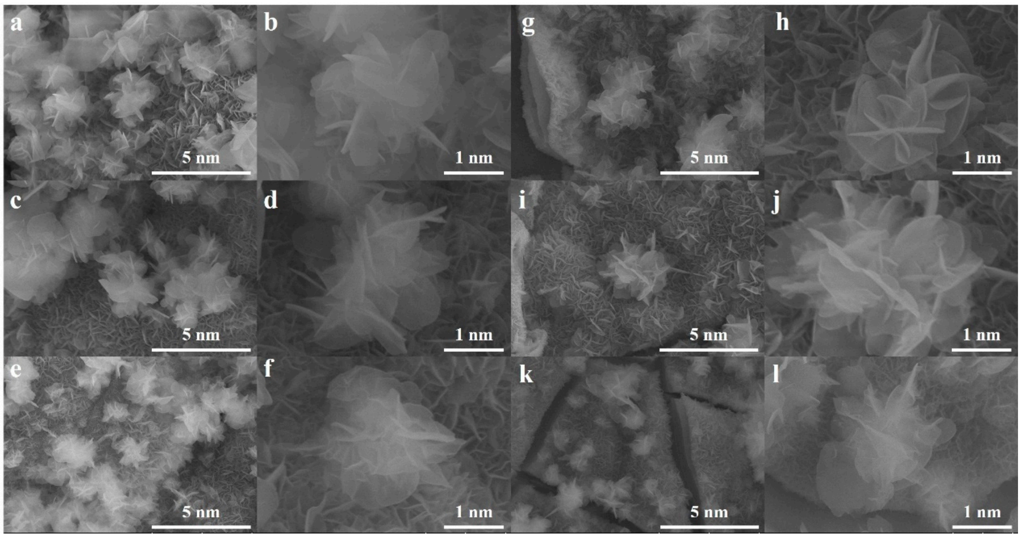

Figure S1. The SEM images of (a,b) NiFe-LDH; (k,l) S-NiFe-LDH-10; (c,d) S-NiFe-LDH-30; (e,f) S-NiFe-LDH-60; (g,h) S-NiFe-LDH-120 and (i,j) S-NiFe-LDH-240.

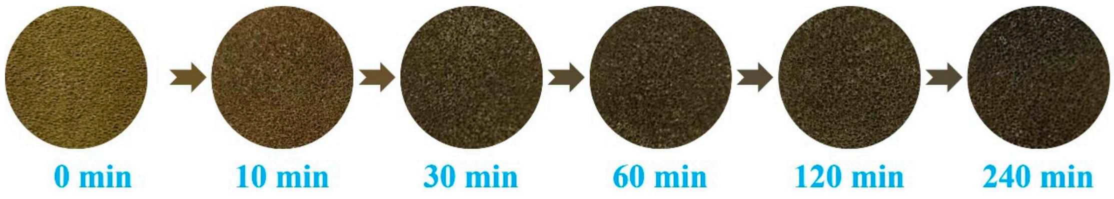

Figure S2. The digital photograph of time-dependent color evolution of NiFe-LDH during sulfur modification in Na_2S solution.

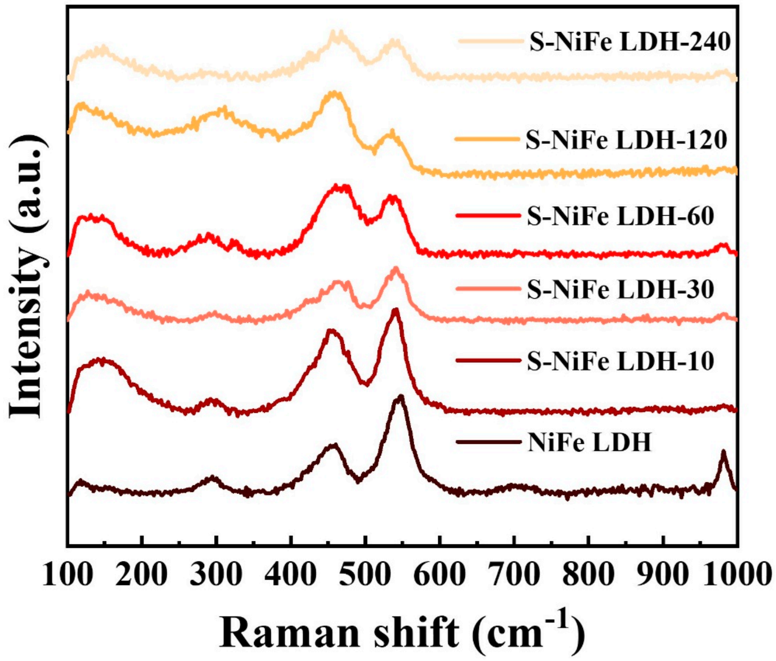

Figure S3. Raman spectra of NiFe-LDH and S-NiFe-LDH-t.

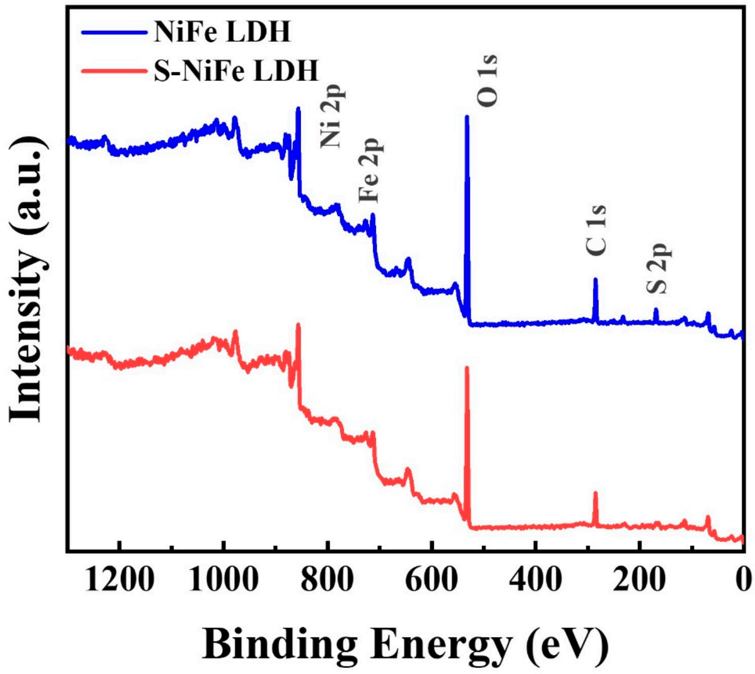

Figure S4. XPS survey spectrum of NiFe-LDH and S-NiFe-LDH.

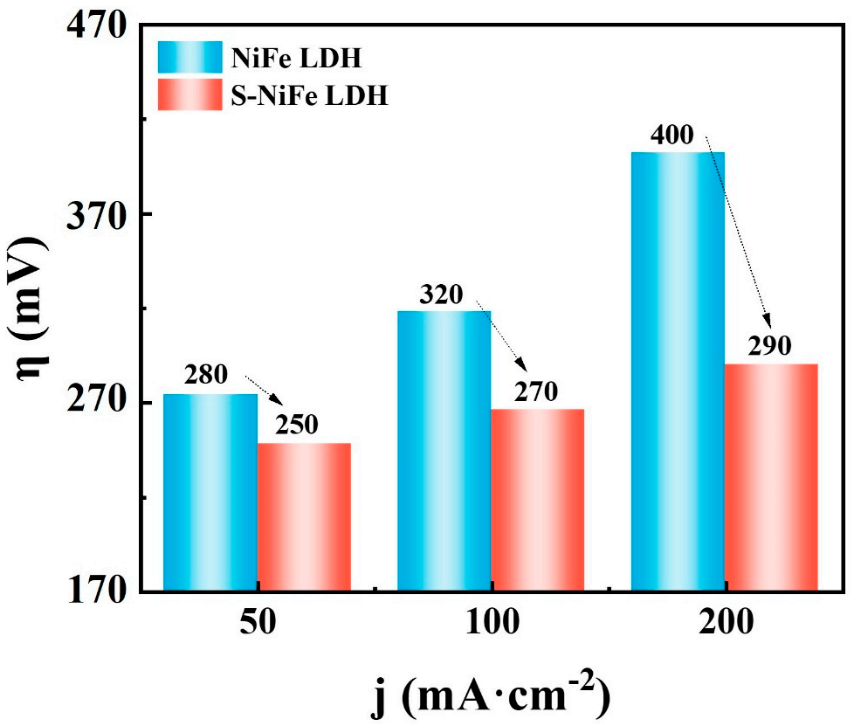

Figure S5. The corresponding overpotentials at 50, 100, and 200 mA·cm^{-2} of NF, NiFe-LDH, and S-NiFe-LDH.

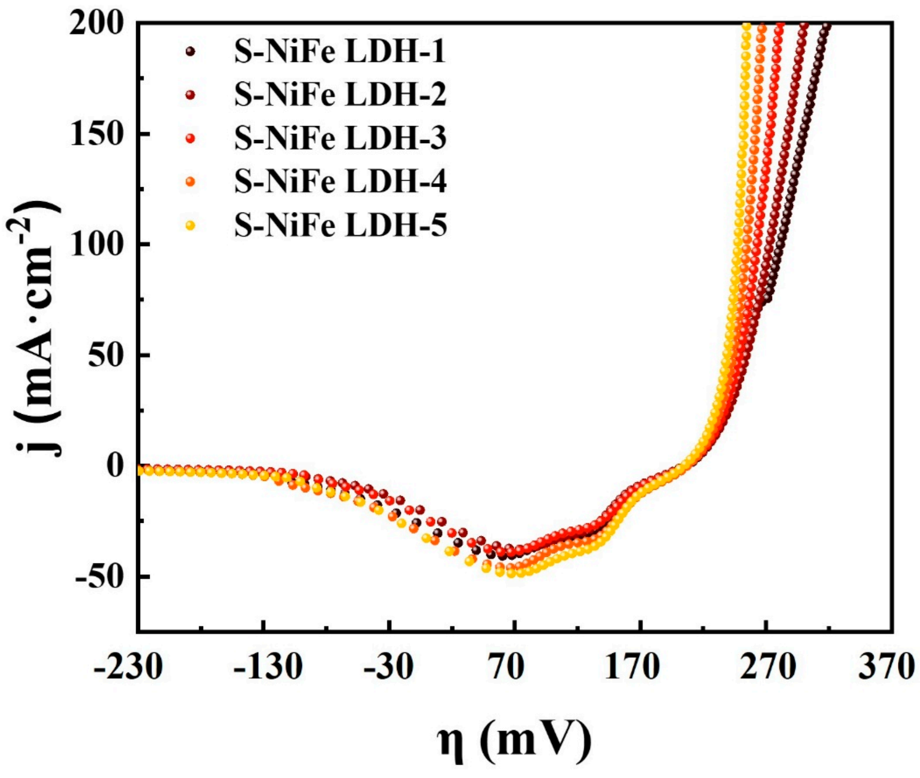

Figure S6. LSV curves of S-NiFe-LDH-t samples.

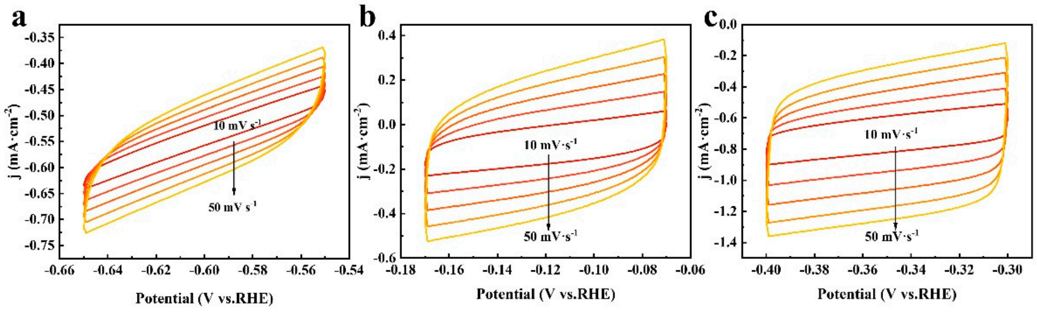

Figure S7. CV curves of (a) NF, (b) NiFe-LDH, and (f) S-NiFe-LDH in a nonfaradaic region with scan rate of 10-50 mV s^{-1}.

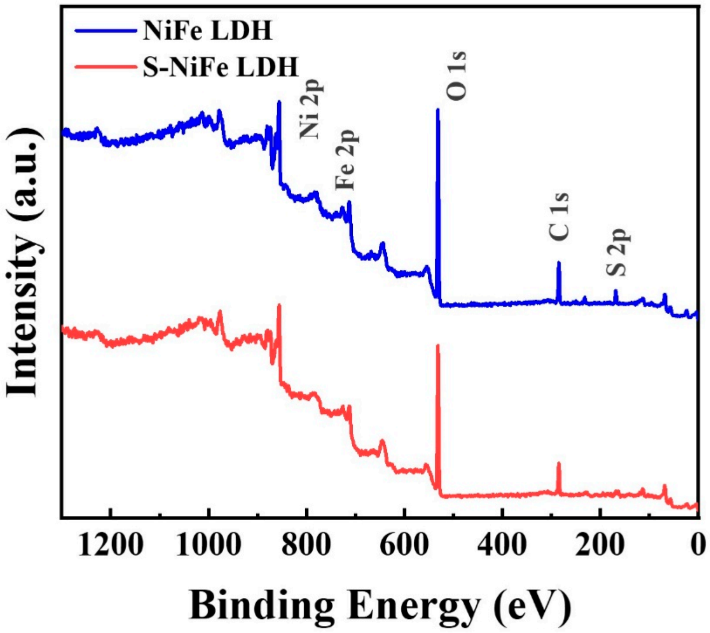

Figure S8. XPS survey spectrum of S-NiFe-LDH before and after OER stability reactions.

Table S1. Comparison of Ni-based catalyst reported recently for OER electrocatalytic performance.

Electrocatalysts	Electrolyte	η_{100} (mV)	Tafel (mV·dec^{-1})	Reference
S-NiFe-LDH	1 M KOH	270	22.3	This work
la-S-NiFe-LDH/NFF	1 M KOH	245	55.8	[6]
NiFe-LDH/$FeNi_2S_4$/IF	1 M KOH	310	51.0	[7]
SNiFe@NF	6 M KOH natural Seawater	261	20.14	[8]
S-$NiMoO_4$@NiFe-LDH	1 M KOH + 0.5 M NaCl	273	90	[9]
S-NiFe-LDH/MXene@NF	1 M KOH	264[a]	60.1	[10]
NiFe-LDH-S350	1 M KOH + 0.5 M NaCl	296	131	[11]
S-$NiFeSe_2$	1 M KOH + Seawater	367	32	[12]
Ni_2Fe-LDH/$FeNi_2S_4$/NF	1 M KOH	240	29.4	[13]
NiFe-LDH-Ni_3S_2	1 M KOH	240	43.5	[14]
S-(Ni,Fe)OOH	1 M KOH	281	48.9	[15]
NFF O-R 20 s	1 M KOH	308	39.85	[16]
(Ni,Fe)S_2@Ti_3C_2	1 M KOH	266[b]	26	[17]

a: η_{50} = 264 mV; b: η_{500} = 266 mV.

Table S2. Summary of electrocatalytic performance for OER catalysts in seawater.

Catalyst	Electrolyte	Performance	Stability	Refs.
S-NiFe-LDH	Alkaline seawater	270 mV@100 mA·cm^{-2}	200 h@100 mA·cm^{-2}	This work
NiMoN@NiFeN	Alkaline seawater	307 mV@100 mA·cm^{-2}	100 h@ 100 mA·cm^{-2}	[18]
$Na_2Co_{1-x}Fe_xP_2O_7$	Alkaline seawater	~ 300 mV@10 mA·cm^{-2}	50 h@ 10 mA·cm^{-2}	[19]
Ni_2P-Fe_2P/NF	Alkaline seawater	305 mV@10 mA·cm^{-2}	36 h@ 100 mA·cm^{-2}	[20]
Au-Gd-Co_2B@TiO_2	Alkaline seawater	280 mV@100 mA·cm^{-2}	200 h@ 100 mA·cm^{-2}	[21]
Co-Fe_2P	Alkaline seawater	266 mV@10 mA·cm^{-2}	22 h@ 100 mA·cm^{-2}	[22]
CoP_x@FeOOH	Alkaline seawater	235 mV@10 mA·cm^{-2}	80 h@ 100 mA·cm^{-2}	[23]
NiCoP/NiCo-LDH@NF	Alkaline seawater	350 mV@10 mA·cm^{-2}	50 h@ 15 mA·cm^{-2}	[24]
NiCoS	Alkaline seawater	280 mV@10 mA·cm^{-2}	100 h@ 100 mA·cm^{-2}	[25]
S-doped Ni/Fe	Alkaline seawater	300 mV@10 mA·cm^{-2}	100 h@ 100 mA·cm^{-2}	[15]
Fe@Ni–Co@NF	Alkaline seawater	246 mV@50 mA·cm^{-2}	12 h@ 1000 mA·cm^{-2}	[26]
Ni_3FeN@C/NF	Alkaline seawater	314 mV@100 mA·cm^{-2}	100 h@ 100 mA·cm^{-2}	[27]
0.5Fe-$NiCo_2O_4$	Alkaline seawater	293 mV@10 mA·cm^{-2}	20 h@ 1550 mV	[28]

References

1. Ning, M.; Wu, L.; Zhang, F.; Wang, D.; Song, S.; Tong, T.; Bao, J.; Chen, S.; Yu, L.; Ren, Z. One-step spontaneous growth of NiFe layered double hydroxide at room temperature for seawater oxygen evolution. *Mater. Today Phys.* **2021**, *19*, 100419.
2. Li, Z.; Lin, G.; Wang, L.; Lee, H.; Du, J.; Tang, T.; Ding, G.; Ren, R.; Li, W.; Cao, X.; et al. Seed-assisted formation of NiFe anode catalysts for anion exchange membrane water electrolysis at industrial-scale current density. *Nat. Catal.* **2024**, *7*, 944–952.
3. Kresse, G.; Furthmüller, J.J.P.R.B. Efficient iterative schemes for ab initio total-energy calculations using a plane-wave basis set. *Phys. Rev. B* **1996**, *54*, 11169.
4. Han, L.; Jing, F.; Zhang, J.; Luo, X.-Z.; Zhong, Y.-L.; Wang, K.; Zang, S.-H.; Teng, D.-H.; Liu, Y.; Chen, J.; et al. Environment friendly and remarkably efficient photocatalytic hydrogen evolution based on metal organic framework derived hexagonal/cubic In_2O_3 phase-junction. *Appl. Catal. B Environ.* **2021**, *282*, 119602.
5. Dai, R.; Zeng, J.; Qian, P.; Zhang, S.; Li, S.; El-Bahy, Z.M.; Hu, H.; Zhou, Y.; Xu, X. Influence of Divalent Transition-Metal Cations on the Oxygen Evolution Reaction Activity of Layered Double Hydroxide Nanosheets in Seawater Electrolysis. *ACS Appl. Mater. Interfaces* **2026**, *18*, 1478–1489.
6. Song, S.; Wang, Y.; Tian, P.; Zang, J. Activating lattice oxygen in local amorphous S-modified NiFe-LDH ultrathin nanosheets toward superior alkaline/natural seawater oxygen evolution. *J. Colloid Interface Sci.* **2025**, *677*, 853–862.
7. Ai, L.; Tian, Y.; Xiao, T.; Zhang, J.; Zhang, C.; Jiang, J. Energy-saving hydrogen production from sulfion oxidation-hybrid seawater splitting enabled by superwettable corrosion-resistant NiFe layered double hydroxide/$FeNi_2S_4$ heterostructured nanoarrays. *J. Colloid Interface Sci.* **2024**, *673*, 607–615.
8. Sun, S.; Zhao, Z.; Chen, F.; Wang, S.; Wang, Y.; Yang, X.; Song, S.; Zhang, Y.; Wang, T.; Yuan, Y.; et al. Synergistic enhancement of bifunctional activity and stability of seawater electrolysis by in situ etching and concentration strategies. *J. Alloys Compd.* **2024**, *1004*, 175634.
9. Wang, H.; Chen, L.; Tan, L.; Liu, X.; Wen, Y.; Hou, W.; Zhan, T. Electrodeposition of NiFe-layered double hydroxide layer on sulfur-modified nickel molybdate nanorods for highly efficient seawater splitting. *J. Colloid Interface Sci.* **2022**, *613*, 349–358.
10. Liu, Y.; Hong, X.; Liu, X.; Chen, W.; Tang, J. Electrolysis of Seawater: An Effective Path to Sustainable Hydrogen Production with Sulfur-Doped NiFe-LDH/MXene@NF Electrodes. *ACS Sustain. Chem. Eng.* **2024**, *12*, 11520–11530.
11. Jung, S.Y.; Kang, S.; Kim, K.M.; Mhin, S.; Kim, J.C.; Kim, S.J.; Enkhtuvshin, E.; Choi, S.; Han, H. Sulfur-incorporated nickel-iron layered double hydroxides for effective oxygen evolution reaction in seawater. *Appl. Surf. Sci.* **2021**, *568*, 150965.
12. Chen, X.; Yu, Y.; Han, X.; Wang, H.; Hua, Y.; Wu, D.; Deng, P.; Xiao, J.; Tian, X.; Li, J. Introducing sulfur to nickel-iron selenide for high-efficiency alkaline seawater electrolysis. *Sci. China Chem.* **2024**, *67*, 2747–2754.
13. Tan, L.; Yu, J.; Wang, C.; Wang, H.; Liu, X.; Gao, H.; Xin, L.; Liu, D.; Hou, W.; Zhan, T. Partial Sulfidation Strategy to NiFe-LDH@$FeNi_2S_4$ Heterostructure Enable High-Performance Water/Seawater Oxidation. *Adv. Funct. Mater.* **2022**, *32*, 2200951.
14. Zhang, Z.-H.; Yu, Z.-R.; Zhang, Y.; Barras, A.; Addad, A.; Roussel, P.; Tang, L.-C.; Naushad, M.; Szunerits, S.; Boukherroub, R. Construction of desert rose flower-shaped NiFe-LDH-Ni_3S_2 heterostructures via seawater corrosion engineering for efficient water-urea splitting and seawater utilization. *J. Mater. Chem. A* **2023**, *11*, 19578–19590.

15. Yu, L.; Wu, L.; McElhenny, B.; Song, S.; Luo, D.; Zhang, F.; Yu, Y.; Chen, S.; Ren, Z. Ultrafast room-temperature synthesis of porous S-doped Ni/Fe (oxy)hydroxide electrodes for oxygen evolution catalysis in seawater splitting. *Energy Environ. Sci.* **2020**, *13*, 3439–3446.
16. Wu, H.; Zhang, Q.; Liu, Q.; Li, M.; Liu, J.; Fu, J.; Wang, E. 20 Seconds to fabricate high-performance NiFe-based anode for seawater electrolysis via bidirectional pulse current method. *Chem. Eng. J.* **2024**, *498*, 155435.
17. Wang, J.; Liu, Y.; Yang, G.; Jiao, Y.; Dong, Y.; Tian, C.; Yan, H.; Fu, H. MXene-Assisted NiFe sulfides for high-performance anion exchange membrane seawater electrolysis. *Nat. Commun.* **2025**, *16*, 1319.
18. Yu, L.; Zhu, Q.; Song, S.; McElhenny, B.; Wang, D.; Wu, C.; Qin, Z.; Bao, J.; Yu, Y.; Chen, S.; et al. Non-noble metal-nitride based electrocatalysts for high-performance alkaline seawater electrolysis. *Nat. Commun.* **2019**, *10*, 5106.
19. Song, H.J.; Yoon, H.; Ju, B.; Lee, D.-Y.; Kim, D.-W. Electrocatalytic Selective Oxygen Evolution of Carbon-Coated Na2Co1–xFexP2O7 Nanoparticles for Alkaline Seawater Electrolysis. *ACS Catal.* **2019**, *10*, 702–709.
20. Wu, L.; Yu, L.; Zhang, F.; McElhenny, B.; Luo, D.; Karim, A.; Chen, S.; Ren, Z. Heterogeneous Bimetallic Phosphide Ni_2P-Fe_2P as an Efficient Bifunctional Catalyst for Water/Seawater Splitting. *Adv. Funct. Mater.* **2020**, *31*, 2006484.
21. Haq, T.U.; Pasha, M.; Tong, Y.; Mansour, S.A.; Haik, Y. Au nanocluster coupling with Gd-Co2B nanoflakes embedded in reduced TiO_2 nanosheets: Seawater electrolysis at low cell voltage with high selectivity and corrosion resistance. *Appl. Catal. B Environ.* **2022**, *301*, 120836.
22. Wang, S.; Yang, P.; Sun, X.; Xing, H.; Hu, J.; Chen, P.; Cui, Z.; Zhu, W.; Ma, Z. Synthesis of 3D heterostructure Co-doped Fe_2P electrocatalyst for overall seawater electrolysis. *Appl. Catal. B Environ.* **2021**, *297*, 120386.
23. Wu, L.; Yu, L.; McElhenny, B.; Xing, X.; Luo, D.; Zhang, F.; Bao, J.; Chen, S.; Ren, Z. Rational design of core-shell-structured CoP@FeOOH for efficient seawater electrolysis. *Appl. Catal. B Environ.* **2021**, *294*, 120256.
24. Wu, Y.; Tian, Z.; Yuan, S.; Qi, Z.; Feng, Y.; Wang, Y.; Huang, R.; Zhao, Y.; Sun, J.; Zhao, W.; et al. Solar-driven self-powered alkaline seawater electrolysis via multifunctional earth-abundant heterostructures. *Chem. Eng. J.* **2021**, *411*, 128538.
25. Wang, C.; Zhu, M.; Cao, Z.; Zhu, P.; Cao, Y.; Xu, X.; Xu, C.; Yin, Z. Heterogeneous bimetallic sulfides based seawater electrolysis towards stable industrial-level large current density. *Appl. Catal. B Environ.* **2021**, *291*, 120071.
26. Jadhav, A.R.; Kumar, A.; Lee, J.; Yang, T.; Na, S.; Lee, J.; Luo, Y.; Liu, X.; Hwang, Y.; Liu, Y.; et al. Stable complete seawater electrolysis by using interfacial chloride ion blocking layer on catalyst surface. *J. Mater. Chem. A* **2020**, *8*, 24501–24514.
27. Wang, B.; Lu, M.; Chen, D.; Zhang, Q.; Wang, W.; Kang, Y.; Fang, Z.; Pang, G.; Feng, S. NixFeyN@C microsheet arrays on Ni foam as an efficient and durable electrocatalyst for electrolytic splitting of alkaline seawater. *J. Mater. Chem. A* **2021**, *9*, 13562–13569.
28. Yang, J.; Wang, Y.; Yang, J.; Pang, Y.; Zhu, X.; Lu, Y.; Wu, Y.; Wang, J.; Chen, H.; Kou, Z.; et al. Quench-Induced Surface Engineering Boosts Alkaline Freshwater and Seawater Oxygen Evolution Reaction of Porous $NiCo_2O_4$ Nanowires. *Small* **2021**, *18*, 2106187.

Disclaimer/Publisher's Note: The statements, opinions and data contained in all publications are solely those of the individual author(s) and contributor(s) and not of MDPI and/or the editor(s). MDPI and/or the editor(s) disclaim responsibility for any injury to people or property resulting from any ideas, methods, instructions or products referred to in the content.

https://doi.org/10.3390/xxxxx
